# Numb is required to prevent p53-dependent senescence following skeletal muscle injury

**DOI:** 10.1038/ncomms9528

**Published:** 2015-10-27

**Authors:** Isabelle Le Roux, Julie Konge, Laurent Le Cam, Patricia Flamant, Shahragim Tajbakhsh

**Affiliations:** 1 Department of Developmental and Stem Cell Biology, Stem Cells and Development, CNRS URA 2578, Institut Pasteur, 25 rue du Dr Roux, Paris 75015, France; 2 Human Histopathology and Animal Models, Institut Pasteur, 25 rue du Dr Roux, Paris 75015, France; 3 Molecular Basis of Carcinogenesis, Institut de Recherche en Cancérologie de Montpellier, 208 rue des Apothicaires, Montpellier, cedex 5 34298, France

## Abstract

Regeneration relies on coordinated action of multiple cell types to reconstitute the damaged tissue. Here we inactivate the endocytic adaptor protein Numb in skeletal muscle stem cells prior to chronic or severe muscle injury in mice. We observe two types of senescence in regenerating muscle; a transient senescence in non-myogenic cells of control and *Numb* mutant mice that partly depends on *INK4a/ARF* activity, and a persistent senescence in myogenic cells lacking Numb. The senescence levels of *Numb*-deficient muscle is reduced to wild type levels by an anti-oxidant treatment or *p53* ablation, resulting in functional rescue of the regenerative potential in *Numb* mutants. *Ex vivo* experiments suggest that *Numb*-deficient senescent cells recruit macrophages to sustain inflammation and drive fibrosis, two hallmarks of the impaired muscle regeneration in *Numb* mutants. These findings provide insights into previously reported developmental and oncogenic senescence that are also differentially regulated by p53.

## Abstract (web summary)

Regeneration of skeletal muscle relies on the function of muscle satellite cells. Here, Le Roux *et al*. show that the endocytic adaptor protein Numb promotes skeletal muscle regeneration after injury by preventing a p53-dependent senescence of satellite cells and consequent inflammation and fibrosis.

## Graphical abstract

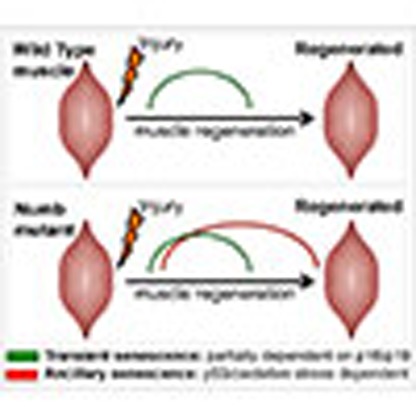

Tissue regeneration is characterised by three distinct overlapping phases including inflammation, tissue reconstruction and remodelling. In skeletal muscle, the paired/homeodomain protein Pax7 is a marker of muscle stem (satellite) cells and Pax7-positive cells are critically required for muscle regeneration^1^,^2^,^3^. Following injury, satellite cells are activated, they proliferate, and some resulting myoblasts differentiate and fuse to form new myofibers, whereas a subset return to quiescence and replenish the stem cell niche^4^,^5^. During the expansion of satellite cells, muscle-resident fibroblasts proliferate, provide pro-differentiation signals to myoblasts, and secrete extracellular matrix thereby stabilizing the tissue^6^,^7^. Concomitantly to myogenesis, angiogenesis stimulates myogenic growth^4^,^5^. In addition, the inflammatory response that is mediated through the action of macrophages is necessary to repair damaged tissues. Communication between these distinct cell types is crucial during the process of regeneration, as sustained inflammation drives aberrant fibrosis and contributes to pathology^8^.

Senescent cells act in paracrine and via their secretome induce a local inflammatory response leading to their elimination by phagocytosis. Thus, cellular senescence is a mechanism contributing to tissue remodelling, particularly during tumour formation, organogenesis and as reported recently, during the process of wound healing^9^,^10^,^11^,^12^,^13^,^14^. Paradoxically, senescent cells can be beneficial and detrimental for tissue constitution^15^. Senescent cells share common features such as an irreversible cell cycle arrest, a change in morphology, senescence-associated heterochromatin foci, and a senescence-associated secretory phenotype^15^. In addition, senescent cells can be identified by histochemical detection of β-galactosidase activity under acidic conditions, called senescence-associated β-galactosidase activity (SAβGal; ref. 16). Multiple stresses induce senescence, which is regulated mainly by the tumour suppressors p16, p19, p53 and Rb, as well as the cyclin-dependent kinase inhibitors p21 and p27 (ref. 15).

Studies have focused mainly on the beneficial action of non-myogenic cells during muscle regeneration, yet it remains unclear to what extent satellite cells and their committed progeny communicate with their environment. The endocytic adaptor Numb possesses multiple protein–protein interaction domains that confer pleiotropic functions including modulation of Notch, Shh and Wnt signalling^17^,^18^,^19^,^20^. Thus, to explore the possibility that Numb can mediate myogenic cell communication in skeletal muscle, we examined the function of this protein specifically in the myogenic lineage following muscle injury where it was reported to control different steps during muscle regeneration^21^,^22^,^23^. We show that deletion of *Numb/Numbl* in satellite cells prior to injury lead to impaired regeneration marked by increased inflammation and fibrosis. Importantly we unveiled two types of senescence during regeneration; a transient senescence in non-myogenic cells in control and *Numb* mutant mice, which is partially dependent on *Ink4A/ARF* activity, and a persistent senescence in myogenic cells, exclusively in *Numb* mutant mice. The latter depends on p53 and is rescued by the administration of anti-oxidant. *In vivo* and *ex vivo* experiments further showed that *Numb* mutant-specific senescent cells are responsible for the impaired regeneration phenotype.

## Results

### Impaired regeneration in *Numb* mutants following acute injury

**Figure 1:**
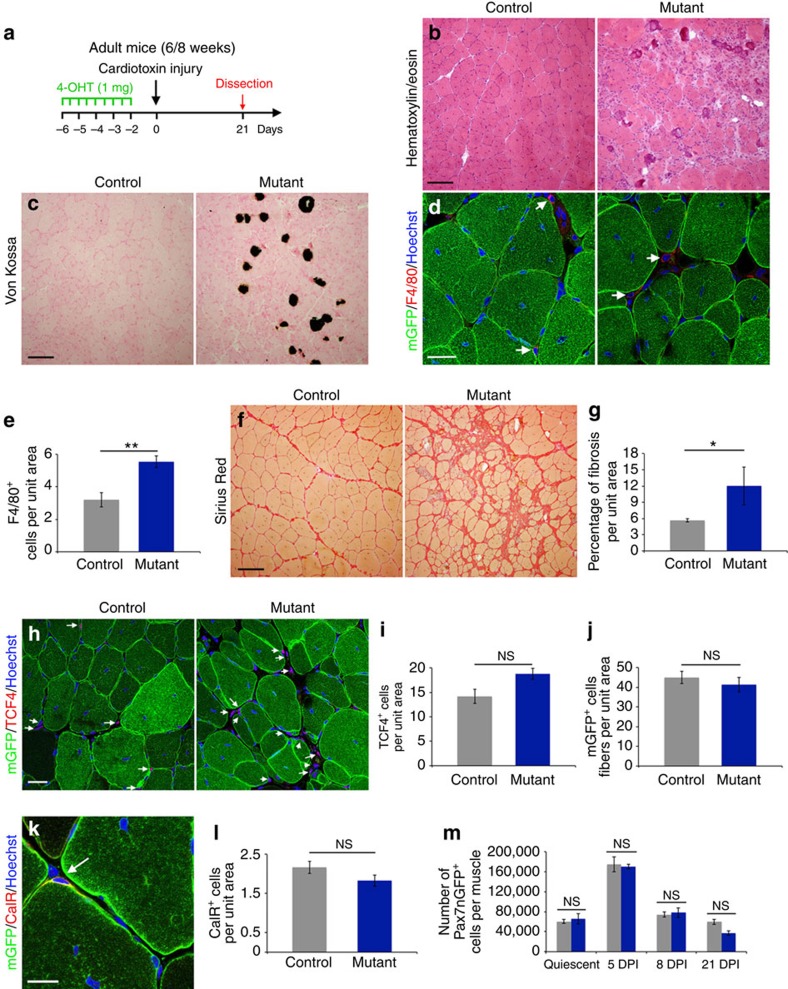
Incomplete regeneration following deletion of *Numb:Numbl* in satellite cells. (**a**) Strategy used to study TA muscle regeneration in the context of *Numb:Numbl* depleted satellite cells. Data were collected from transverse TA muscle cryosections of adult mice. (**b**) Histological staining with H&E. (**c**) Histological staining with Von Kossa. Dark precipitates reveal calcium deposits, a sign of chronic inflammation. (**d**) Immunofluorescence staining for macrophages (arrows) using anti-F4/80 antibody. (**e**) Quantification of the number of macrophages in (**d**); control: *n*=5 mice, 3.2±0.4 cells per unit area; mutant: *n*=5 mice, 5.6±0.4 cells per unit area; Mann-Whitney (MW) test *P*=0.079. (**f**) Histological staining with Sirius Red. (**g**) Quantification of fibrosis based on Sirius Red histology in (**f**); control: *n*=5 TA, 5 mice, 5.6±0.3% fibrosis per unit area; mutant: *n*=5 TA, 5 mice, 12±3.5% fibrosis per unit area; MW test *P*=0.0397. (**h**) Immunofluorescence staining for fibroblasts (arrows) using anti-TCF4 antibody. (**i**) Quantification of the number of fibroblasts in (**h**); control: *n*=4 mice, 14.2±1.4 cells per unit area; mutant: *n*=4 mice, 18.8±1.12 cells per unit area; MW test *P*=0.0571. (**j**) Quantification of the numbers of mGFP^+^ fibres; control: *n*=3 TA, 3 mice mice, 45±3.1 fibres per unit area; mutant: *n*=6 TA, 3 mice, 41.4±3.7 fibres per unit area; MW test *P*=0.2619. (**k**) Immunofluorescence staining for quiescent satellite cells (arrows) using anti-Calcitonin receptor antibody. (**l**) Quantification of quiescent satellite cells in (**k**); control: *n*=4 mice, 2.17±0.15 cells per unit area; mutant: *n*=4 mice, 1.83±0.14 cells per unit area; MW test *P*=0.20. (**m**) Quantification of the number Pax7nGFP^+^ cells in one diaphragm for quiescent cells; control: *n*=8 mice, 60,376±4,038; mutant: *n*=7 mice, 65,663±10,106; MW *P*=0.6943) and in 2 TA for activated cells (control 5 DPI: *n*=3 mice, 174,880±15,190; mutant 5 DPI: *n*=3 mice, 170,490±4,747, MW *P*>0.99; control 8 DPI: *n*=12 mice, 74,306±5,002; mutant 8 DPI: *n*=7 mice, 77,852±9,562, MW *P*=0.9671; control 21 DPI: *n*=3 mice, 59,935±5,284; mutant 21 DPI: *n*=4, 36,867±4,276, MW *P*=0.0571). Quantifications are presented as mean±s.e.m. Scale bars **b**,**c**,**f**: 100 μM; **d**,**h**: 25 μM; **k**: 20 μM. NS, not significant.

Numb is widely expressed in different cell types in the muscle and we observed that this protein is expressed in about 85% of both quiescent and *ex vivo* activated satellite cells (Supplementary Fig.1a–c). To investigate the function of Numb specifically in myogenic cells, we performed a conditional inactivation of *Numb* using an inducible *Tg:Pax7-Cre*^*ERT2*^ (hereafter *Tg:Pax7CT2*)^24^ in satellite cells in the context of a constitutive mutant for its paralog *Numbl*^25^ (Supplementary Fig. 1b). Following 4-hydroxytamoxifen (4-OHT) injections, adult *Tg:Pax7CT2;Numb*^*F/F*^*;Numbl*^*Δ/Δ*^ mice (hereafter *Numb:Numbl* or mutant) were indistinguishable from adult control mice; 64% of their satellite cells lacked Numb expression at T0, and after 40 h in culture (Supplementary Fig. 1c). We then used *R26*^*mT-mG*^ reporter mice^26^ to isolate Numb depleted cells. Among the recombined mGFP^+^ cells, 62% (*n*=3) were depleted for Numb protein (data not shown). The *Tibialis anterior* (TA) muscle was injured with the snake venom cardiotoxin, collected and analysed at different time points during regeneration. Importantly, isolated mGFP^+^ cells displayed a persistent decrease in *Numb* transcript levels by about 50% compared with controls at all time points examined during homeostasis, regeneration, and after muscle recovery (Supplementary Fig. 1d). Strikingly, *Numb* transcript levels in controls increased late in regeneration, suggesting a function for Numb at these stages. At 21 days post-injury (DPI), histological analysis of controls showed centrally localised myonuclei, a hallmark of regenerating myofibers (Fig. 1a, b). In contrast, mutant TA muscles displayed a highly perturbed morphology, including a high number of interstitial cells and heterogenous myofiber sizes, the latter persisting at 60 DPI (Fig. 1b; Supplementary Fig. 1f). Moreover, an inflammatory phenotype was noted by the presence of calcium deposits (Von Kossa staining) and a 1.7-fold increase in the number of macrophages (F4/80^+^ cells) in mutant TA muscles compared with controls (Fig. 1c–e). In addition, Sirius Red staining on TA muscle revealed increased fibrosis in mutants that was accompanied by a slight increase in the number of fibroblast-like (Tcf4^+^) cells (Fig. 1f–h). Taken together these results show that in the absence of Numb/Numbl, muscle regeneration was compromised.

Using *in vivo* and *ex vivo* approaches, we noted that the ability of mutant cells to proliferate, generate new myofibers, return to quiescence following injury, and differentiate was not overtly compromised in the absence of Numb/Numbl (Fig. 1j–m; Supplementary Figs 1c and 2a–m; see also Supplementary Fig. 3a–d). Notably, the number of Pax7^+^ cells and the relative levels of *Pax7* transcripts in mGFP^+^ cells were not significantly changed in mutant muscle compared with control muscle at homeostasis and during regeneration (Fig. 1m; Supplementary Fig. 1e). These data contrast with those reported previously showing decreased satellite cell proliferation after injury when *Numb* was deleted in the Pax7 lineage from embryonic stages to adulthood^23^. Those *Numb* mutant mice were smaller at 1 month of age and they exhibited smaller fiber size suggesting that Numb plays an important additional role during developmental myogenesis or perinatally. Collectively, these observations suggest that Numb has distinct functions in proliferating muscle progenitors during development, perinatally and in adult satellite cells, as it is the case for Pax7^27^,^28^,^29^. In addition, the absence of an overt differentiation phenotype strongly suggests that Numb does not inhibit Notch signalling (Supplementary Fig. 2a–i)^24^,^30^. This point was supported by the observation that the relative levels of Notch target transcripts (*Hey1* and *HeyL*) in isolated mGFP^+^ cells were similar in mutants and controls during homeostasis, and at different time points following injury (Supplementary Fig. 2n and o).

### Lack of Numb exacerbates the muscle dystrophic phenotype

**Figure 2:**
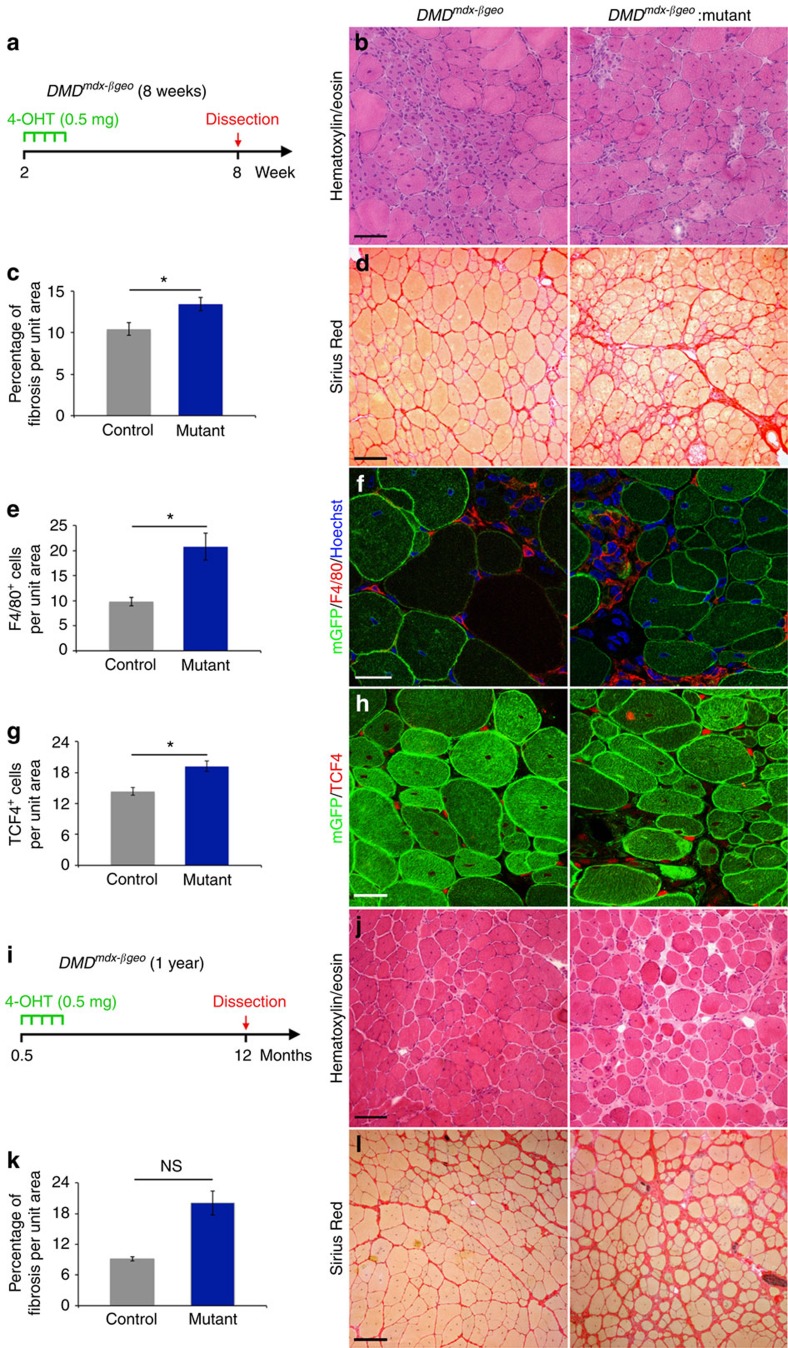
Loss of Numb/Numbl exacerbates the dystrophic muscle phenotype. Data were collected from transverse TA muscle cryosections from *DMD*^*mdx-βgeo*^ mice. (**a**) Scheme of the experiments. (**b**) Histological staining with H&E. (**c**) Quantification of fibrosis in mutants and controls in 8-week-old *DMD*^*mdx-βgeo*^ mice based on Sirius Red histology (control: *n*=8 TA, 5 mice, 10.4±0.8% fibrosis per unit area; mutant: *n*=13 TA, 7 mice, 13.5±0.8% fibrosis per unit area; MW *P*=0.0303). (**d**) Histological staining with Sirius Red. (**e**) Quantification of F4/80^+^ cells (control: *n*=4 mice, 9.8±0.9 cells per unit area; mutant: *n*=5 mice, 20.8±2.7 cells per unit area; MW *P*=0.0159). (**f**) Immunofluorescence staining for macrophages using anti-F4/80 antibody. (**g**) Quantification of Tcf4^+^ (control: *n*=4 mice, 14.3±0.7 cells per unit area; mutant: *n*=4 mice, 19.2±1.0 cells per unit area; MW *P*=0.0286). (**h**) Immunofluorescence staining for fibroblasts using anti-Tcf4 antibody. (**i**) Scheme of the experiments. (**j**) Histological staining with H&E. (**k**) Quantification of fibrosis in mutants and controls in 1-year-old *DMD*^*mdx-βgeo*^ mice based on Sirius Red histology and (control: *n*=3 TA, 3 mice, 9.1±0.4% fibrosis per unit area; mutant: *n*=3 TA, 3 mice, 20.1±2.4% fibrosis per unit area; MW *P*=0.10). (**l**) Histological staining with Sirius Red. Quantifications are presented as mean±s.e.m.. Scale bars **b**,**d**,**j**,**l**: 100 μM; **f**,**h**: 25 μM. NS, not significant.

We then focused on a more physiological model of regeneration by examining *DMD*^*mdx-βgeo*^ mice in a *Numb:Numbl* null mutant background. *DMD*^*mdx-βgeo*^ mice constitute a model of muscular dystrophy where dystrophin, a major component of the architecture of the myofiber, is lacking^31^. In this model, myofibers continuously and asynchronously degenerate, and a proportion of satellite cells is randomly activated to repair the damaged fibres^32^. Moreover, *DMD*^*mdx-βgeo*^ mice exhibit chronic inflammation and aberrant fibrosis. Remarkably, at early stages of the disease (8 weeks), *Numb:Numbl:DMD*^*mdx-βgeo*^ mutants displayed an increase in fibrosis compared with *DMD*^*mdx-βgeo*^ mice (Fig. 2a–d). Similarly to the phenotype observed after acute injury, the number of macrophages and fibroblasts in TA muscle was increased significantly in *Numb:Numbl:DMD*^*mdx-βgeo*^ mice compared with *DMD*^*mdx-βgeo*^ mutants (Fig. 2e–h). In 1-year-old mice, increased fibrosis was still observed in *Numb:Numbl:DMD*^*mdx-βgeo*^ mutants compared with *DMD*^*mdx-βgeo*^ mutants, although this was not statistically significant, (Fig. 2i–l). We thus conclude that loss of Numb/Numbl in the myogenic lineage can exacerbate the muscular dystrophic phenotype.

### Self-renewed mutant cells acquire features of senescence

**Figure 3:**
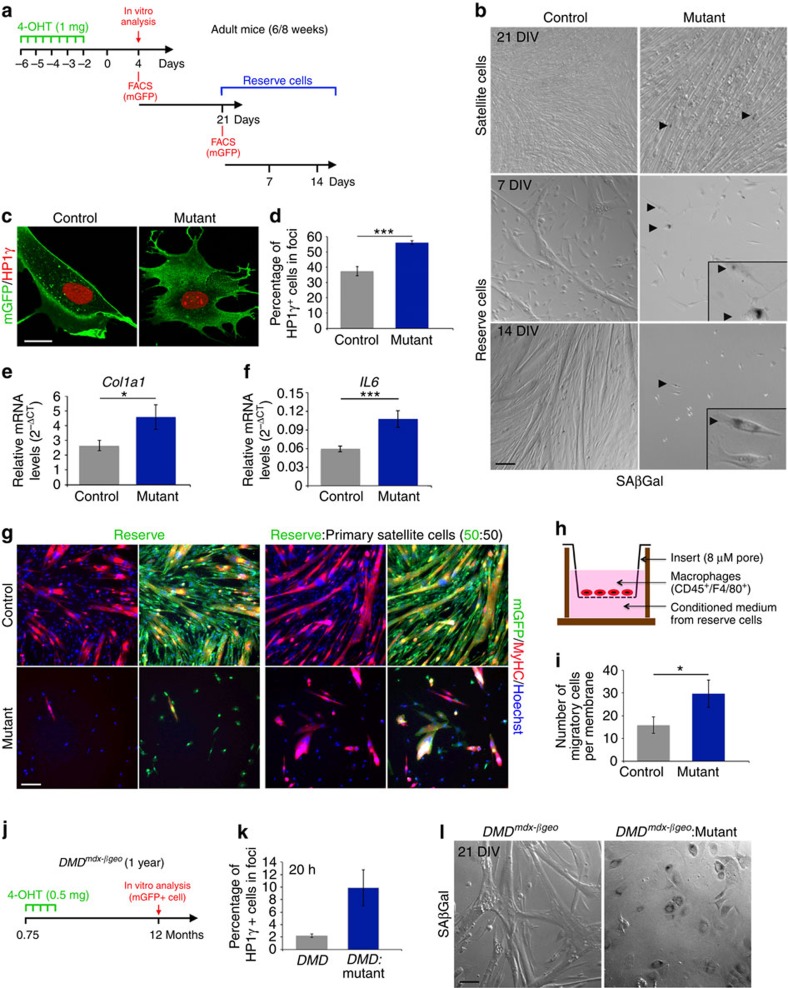
Self-renewed mutant satellite cells acquire senescence properties. (**a**) Strategy used to obtain and examine reserve or self-renewed cells. (**b**) SAβGal staining on confluent myotubes derived from isolated mGFP^+^ satellite cells maintained 21 days *in vitro* (21 DIV) and on replated isolated mGFP^+^ reserve cells after 7 and 14 DIV. Arrowheads point to SAβGal^+^ cells (dark staining). Windows in left centre and bottom pictures represent high magnifications of the main image highlighting SAβGal^+^ cells. Data are representative of 3 independent experiments using 2 controls and 2 mutants for each. (**c**) Immunofluorescence using anti-GFP and anti-HP1γ antibodies on reserve cells 20 h after plating. (**d**) Quantification of the number of self-renewed cells with HP1γ nuclear foci as opposed to uniform staining at 20 h after plating (control *n*=9 mice, 37.4±2.9%; mutant: *n*=9 mice, 56.1±1.2%; MW *P*<0.0001). (**e,f**) Relative levels of *Col1a1* (control *n*=13 mice and mutant *n*=13 mice; MW *P*=0.0207) and *IL6* (control *n*=8 and mutant *n*=8 mice; MW *P*=0.0007) mRNA (2^-ΔCT^) in reserve mutant cells compared with controls. TBP was used as reference gene. (**g**) Immunofluorescence using anti-GFP and anti-Myosin Heavy Chain (MyHC) antibodies on control and mutant reserve cells (mGFP^+^) cultured for 9 DIV either alone or mixed at equal ratio with primary isolated satellite cells (mGFP^−^). Left panels represent for each condition the merge pictures of the right panels. Data are representative of 2 independent experiments using 2 controls and 2 mutants for each. (**h**) Scheme of the migration assay of the macrophages. (**i**) Quantification of the number of macrophages that migrate through the transwell membrane (control *n*=7 mice, 15.8±3.6 cells per membrane; mutant *n*=7 mice, 29.7±6 cells per membrane; MW *P*=0.0186). (**j**) Scheme used to study *DMD*^*mdxβgeo*^ satellite cells *in vitro*. (**k**) Quantification of the number of satellite cells with a nuclear localisation of HP1γ in foci at 20 h after plating (control: *n*=2 mice, 2.2±0.3%; mutant: *n*=2 mice, 9.9±2.9%). (**l**) SAβGal staining on satellite cells isolated from 1-year-old *DMD*^*mdx-βgeo*^ mice and cultured 21 DIV. Note extensitive SAβGal staining in *Numb:Numbl:DMD*^*mdx-βgeo*^ mutant cells compared with controls. Quantifications are presented as mean±s.e.m. Scale bars **b**,**g**,: 50 μM, **c**: 10 μM, **l**: 33 μM.

In light of the above findings, we hypothesized that *Numb:Numbl* mutant satellite cells acquired altered properties at late stages of regeneration during the transition to quiescence or differentiation, resulting in impaired regeneration. To test this notion, we cultured satellite cells and allowed them to fuse and form a network of myotubes. In this model, some cells (called reserve) remain mononucleated and acquire satellite cell properties, including the expression of Pax7 (ref. 33). After 21 days *in vitro* (DIV), cells were dissociated and mGFP^+^ mononucleated cells were isolated (Fig. 3a). As expected, most of these cells expressed Pax7 (88±1.3% Pax7^+^/mGFP^+^ cells; *n*=4 mice), independently of their genotype. Remarkably, 7 days after replating, mutant reserve cells stopped proliferating and displayed altered morphology, in contrast to control cells that continued to proliferate, then fused to form myotubes (Fig. 3b and c). Further analysis showed that some cells in confluent culture of myotubes derived from mutant satellite cells were SAβGal^+^, a marker of senescence, whereas none were observed in control (Fig. 3b). This was also the case after 7 and 14 DIV (Fig. 3b). In parallel, we showed that this phenotype was not due to replicative senescence as the doubling population rates of control and mutant primary satellite cells were comparable (Supplementary Fig. 3c and d).

We then investigated the acquisition of other senescence features by mutant reserve cells. The change of the nuclear localisation of HP1γ from uniform staining to foci illustrates the formation of the senescence-associated heterochromatin foci. The number of reserve cells harbouring HP1γ in foci 20 h post-plating was significantly higher in mutant compared with control cells (Fig. 3c and d). The green fluorescent protein (GFP) immunostaining clearly demarcated the morphology of mutant reserve cells as that described for senescent cells (Fig. 3c). Accordingly, mutant cells expressed significantly higher transcript levels of the myofibroblast marker *Collagen1a1* compared with controls (Fig. 3e). We next tested whether mutant reserve cells could act in paracrine on neighbouring cells. Consistent with this hypothesis, IL6 an identified senescence-associated secretory phenotype component^15^, showed significantly higher transcript levels in mutant reserve cells compared with controls (Fig. 3f). Accordingly, the percentage of reserve cells expressing IL6 protein was significantly higher in mutants compared with controls (Supplementary Fig. 3e–h). Paracrine signalling of reserve cells, was further tested by co-culturing reserve cells (mGFP^+^) at equal ratio with primary wild-type satellite cells (mGFP^−^). After 9 DIV, control reserve cells cultured with primary satellite cells contributed to the formation of myofibers (mGFP^+^/Myosin Heavy Chain^+^; Fig. 3g). In contrast, the density and the differentiation of the co-culture with mutant reserve cells were severely compromised. Fewer cells differentiated and most of these cells were derived from primary wild-type satellite cells (mGFP^−^; Fig. 3g). These observations show that the reserve mutant cells act in paracrine to regulate proliferation and differentiation of adjacent wild-type cells in the co-culture assay. These results raise the possibility that *Numb* mutant-specific senescent cells might recruit, via their secretome, macrophages to induce chronic inflammation. To test this notion, we used a transwell assay to examine the ability of conditioned medium derived from reserve cells to influence the migration of macrophages (Fig. 3h). Following 6 h of culture, the migration of macrophages across the transwell membrane increased when incubated with conditioned medium derived from reserve mutant cells compared with the culture with conditioned medium derived from control reserve cells (Fig. 3h and i). Taken together, these findings show that ablation of Numb/Numbl function in the myogenic lineage results in paracrine signalling that impacts on myogenic and macrophage properties.

We then asked if senescence properties can be identified directly in freshly isolated satellite cells. Satellite cells from 1-year-old *DMD*^*mdx-βgeo*^ mice are expected to have undergone multiple rounds of activation and quiescence cycles, thus resembling cultured reserve cells that had returned to quiescence. Notably, five-fold more cells expressed HP1γ in nuclear foci of *Numb:Numbl*:*DMD*^*mdx-βgeo*^ mutants compared with *DMD*^*mdx-βgeo*^ mice after 20 h in culture (Fig. 3j and k). In addition, SAβGal^+^ cells were detected exclusively with *Numb:Numbl*:*DMD*^*mdx-βgeo*^ myogenic cells after 21 DIV (Fig. 3l). In summary, using multiple assays we show that senescence features are induced cell autonomously in satellite cells in the absence of Numb/Numbl during the course of activation and return to quiescence or differentiation, both *in vivo and ex vivo.*

### Increased senescence in the absence of Numb/Numbl

**Figure 4:**
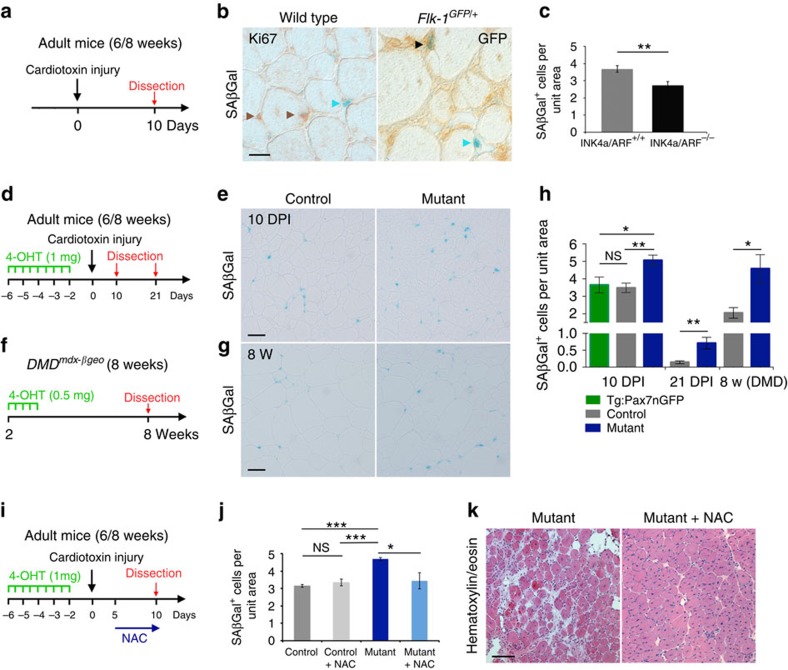
Increased senescence in the absence of Numb/Numbl during muscle regeneration. (**a**) Scheme of the experiments. Data were collected from transverse TA muscle cryosections of adult mice. (**b**) SAβGal staining combined with immunohistochemistry using anti-Ki67 or anti-GFP antibodies on control (left panel) and *Flk-1*^*GFP/+*^(right panel) mice. Brown arrowheads point to Ki67^+^ only cells; blue arrowhead points to SAβGal^+^ only cells and black arrowhead points to GFP^+^/SAβGal^+^ cells. (**c**) Quantification of SAβGal^+^ cells in *INK4a/ARF* mutant mice at 10 DPI (control: *n*=12 TA, 6 mice, 3.7±0.2 cells per unit area; *INK4A/ARF* mutant mice *n*=12 TA, 6 mice, 2.72±0.2 cells per unit area; MW *P*=0.0053). (**d, f**) Schemes of the experiments. (**e, g**) SAβGal staining on transverse TA muscle cryosections. (**h**) Quantification of SAβGal^+^ cells during regeneration in controls and mutants at 10 DPI, 21 DPI, and in 8-weeks old *DMD*^*mdx-βgeo*^ mice. 10 DPI *Tg:Pax7nGFP*: *n*=6 sections, 3 mice, 3.65±0.64 cells per unit area; control: *n*=5 sections, 4 mice, 3.5±0.26 cells per unit area; mutant: *n*=9 sections, 3 mice, 5±0.35 cells per unit area; MW *Tg:Pax7nGFP* versus control *P*=0.7922, *Tg:Pax7nGFP* versus mutant *P*=0.0256, control versus mutant *P*=0.0040. The number of SAβGal^+^ cells at 10 DPI is similar in *Tg:Pax7nGFP* mice compared with controls (*Tg:Pax7CT2;Numb*^*F/+*^*;Numbl*^*Δ/+*^) showing that the tamoxifen treatment or the presence of the Cre recombinase did not interfere with the process. 21 DPI control: *n*=6 sections, 3 mice, 0.14±0.05 cells per unit area; mutant: *n*=6 sections, 4 mice, 0.71±0.21 cells per unit area; MW *P*=0.0043. *DMD*^*mdx-βgeo*^: *n*=6 sections, 3 mice, 2.1± 0.4 cells per unit area; *Numb:Numbl:DMD*^*mdx-βgeo*^: *n*=6 sections, 4 mice, 4.6±1.1 cells per unit area; MW *P*=0.0411. (**i**) Scheme of experiment. (**j**) Quantification of the number of SAβGal^+^ cells (control: *n*=6 sections, 3 mice, 3.2±0.1 cells per unit area; control+NAC: *n*=8 sections, 4 mice, 3.4±0.2 cells per unit area; mutant: *n*=8 sections, 4 mice 4.7±0.1 cells per unit area; mutant+NAC: *n*=8 sections, 4 mice, 3.4±0.5 cells per unit area; MW control versus control+NAC *P*=0.4136, control versus mutant *P*=0.0007, control+NAC versus mutant *P*=0.0006, mutant versus mutant+NAC *P*=0.0148). (**k**) Hematoxylin/eosin staining of mutant TA muscles treated or not with NAC. Quantifications are presented as mean±s.e.m. Scale bars **b**: 50 μM, **e**,**g**,**k**: 100 μM. NS, not significant.

These findings strongly suggest that loss of Numb/Numbl in the Pax7 lineage induces or sustains senescence during regeneration *in vivo*. Senescence was identified as a cellular mechanism of satellite cell aging in geriatric mice (over 28 months) preventing these cells from activation and self-renewal^34^. Therefore, we evaluated senescence *in vivo* in young adult mice to distinguish the process of regeneration from ageing. SAβGal analysis was performed on 10 DPI, 21 DPI and 8-week-old *DMD*^*mdx-βgeo*^ TA muscle from *Numb:Numbl* and control mice. Strikingly, we noted SAβGal^+^ cells in controls, as well as in mutants following acute and chronic injury. These cells were not cycling (Ki67^−^) suggesting that they had undergone senescence (Fig. 4a and b). In the control condition more than half of these cells expressed the endothelial marker Flk-1 (vascular endothelial growth factor-receptor 2) and only a subset of SAβGal^+^ cells expressed the macrophage surface marker F4/80 (Fig. 4b; Supplementary Fig. 4a). Of note, SAβGal^+^ cells did not express Pax7 (Pax7nGFP^−^), Tcf4, nor the pericyte marker NG2 (Supplementary Fig. 4a). Importantly, we observed a 25% reduction in the number of SAβGal^+^ cells at 10 DPI in *Ink4a/ARF* mutant mice compared with controls pointing to a partial role for p16 and/or p19 in the induction of senescence features during regeneration (Fig. 4c). In contrast, no significant difference in the number of SAβGal^+^ cells was observed in mice mutant for either *p21* or *p53* at 10 DPI (Supplementary Fig. 4c and d). These findings are reminiscent of the beneficial action of senescent fibroblasts and endothelial cells during wound healing, and that depends on p16/p21 (ref. 13). Consistent with this notion, two observations suggest that these SAβGal^+^ cells might play a role in tissue remodelling during muscle regeneration. First, the number of SAβGal^+^ cells was transient during early stages of regeneration in controls (about 3.5 cells per unit area at 10 DPI compared to 0.15 cells per unit area at 21 DPI; Fig. 4h). Second, no SAβGal^+^ cells were detected in TA muscle during homeostasis.

Importantly, in all conditions tested, the number of SAβGal^+^ cells was significantly higher in mutant mice compared with controls (Fig. 4d–h). The most notable difference between both genotypes was a five-fold increase in SAβGal^+^ cells at 21 DPI when homeostasis was being restored, representing a third of the total population of quiescent cells (see cell numbers in Fig. 1l and Fig. 4h). The level of reactive oxygen species (ROS) can initiate senescence, and treatments with anti-oxidants delay or prevent cellular senescence^15^. We thus tested if reducing ROS levels during regeneration would alter the number of SAβGal^+^ cells. Mice were exposed to the anti-oxidant N-acetyl cysteine (NAC) from 5–10 DPI and SAβGal^+^ cells were quantified (Fig. 4i). The number of SAβGal^+^ cells was similar in controls with or without NAC treatment. In contrast, treatment with NAC resulted in a reduction in the number of senescent cells in mutants to levels observed in controls (Fig. 4j). Strikingly, histological analysis showed that treatment with NAC resulted in a rescue of the regeneration phenotype and tissue architecture in *Numb:Numbl* null mutants (Fig. 4k). We then investigated if the absence of Numb/Numbl in myogenic cells induces a cell autonomous increase of ROS. To do so, we dissociated muscles at 5 and 10 DPI and incubated cells with the Cell Rox reagent (Supplementary Fig. 4e). At both time points examined, ROS levels in mGFP^+^ cells were comparable between controls and mutants (Supplementary Fig. 4f, g). Thus, the absence of Numb/Numbl does not induce an increase of ROS. Importantly, moderate increased levels of ROS appeared during lineage progression (Supplementary Fig. 4f), and this was shown previously to sensitize foetal myogenic and hematopoietic progenitors cells to differentiation^35^,^36^. It is thus tempting to speculate that the myogenic population affected by the loss of Numb/Numbl corresponds to a sub-population of cells with increased endogenous ROS levels, and that is committed either to differentiate or to return to quiescence. In summary, we identified two types of senescence; one that is transient during muscle regeneration in wild-type mice, and a second type exclusive to *Numb:Numbl* mice, that persists until regeneration was virtually completed. The latter was uniquely rescued by anti-oxidant treatment between 5–10 DPI.

### Loss of Numb induces a p53-dependent senescence

**Figure 5:**
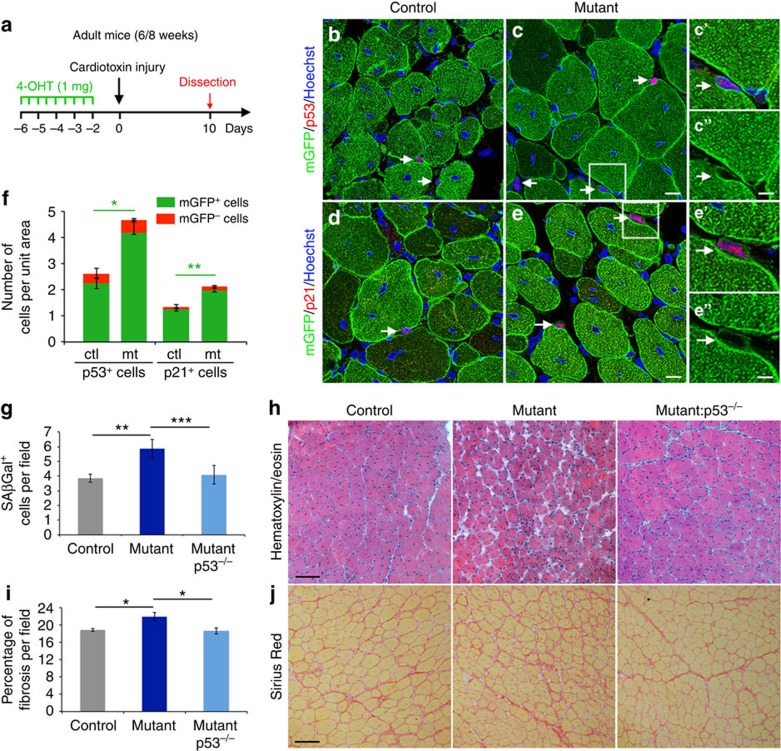
Loss of Numb/Numbl induces senescence in a p53-dependent manner. (**a**) Scheme of the experiment. (**b**–**e**) Immunofluorescence on transverse sections using anti-GFP and anti-p53 (**b**,**c**) or anti-p21 (**d**,**e**) antibodies, arrows point to positive p53 or p21 cells. **c'** and **e'** represent higher magnifications of the area highlighted by a square in **c** and **e**, respectively. **c''** and **e''** are the same images as in **c'** and **e'** without the Hoechst and p53 or p21 staining. (**f**) Quantification of the number of p53^+^ and p21^+^ cells. The number of p53^+^/mGFP^+^ cells increases in mutant compared with control (control: *n*=8 sections, 4 mice, mGFP^+^ 2.24±0.22 cells per unit area, mGFP^−^ 0.37±0.19 cells per unit area; mutant: *n*=8 sections, 4 mice, mGFP^+^ 4.18±0.48 cells per unit area, mGFP^−^ 0.48±0.06 cells per unit area; MW control mGFP^+^ versus mutant mGFP^+^ *P*=0.0145). Similarly, the number of p21^+^/mGFP^+^ cells increases in mutant compared with control (control: *n*=10 sections, 4 mice, mGFP^+^ 1.24±0.06 cells per unit area, mGFP^−^ 0.1±0.06 cells per unit area: mutant: *n*=10 sections, 3 mice, mGFP^+^ 1.96±0.17 cells per unit area, mGFP^−^ 0.15±0.05 cells per unit area; MW control mGFP^+^ versus mutant mGFP^+^ *P*=0.0094). (**g**) Quantification of SAβGal^+^ cells in control, *Numb* and compound *Numb:p53* mutants. The absence of *p53* in *Numb* mutants reduces the number of SAβGal^+^ cells down to control levels (control: *n*=14 sections, 6 mice, 3.85±0.28 cells per unit area; *Numb* mutant: *n*=12 sections, 3 mice, 5.87±0.63 cells per unit area; *Numb:p53* double mutant: *n*=16 sections, 4 mice, 4.08±0.63 cells per unit area; MW control versus *Numb* mutant *P*=0.0010, *Numb* mutant versus *Numb:p53* double mutant *P*=0.0008). (**h**) Hematoxylin/eosin stainings of control and mutant TA muscles. (**i**) Quantification of fibrosis, based on Sirius Red staining (control: *n*=8 TA, 6 mice, 18.90±0.33% fibrosis per unit area; *Numb* null mutant: *n*=6 TA, 3 mice, 21.97±0.92; *Numb:p53* double mutant: *n*=8 TA, 4 mice, 18.63±0.67 cells per unit area; MW control versus *Numb* mutant *P*=0.0205, *Numb* mutant versus *Numb:p53* double mutant *P*=0.0401). (**j**) Sirius Red stainings of control and mutant TA muscles. Quantifications are presented as mean±s.e.m. Scale bars **c**,**e**: 50 μM, **c**”,**e**”: 125 μM; **h**,**j**: 100 μM.

We next aimed at identifying the mediators of senescence that are induced in the absence of Numb/Numbl during muscle regeneration. Immunofluorescence performed on TA muscle revealed that the number of p53^+^ and p21^+^ cells increased significantly in *Numb:Numbl* mutants compared with controls at 10 DPI. Notably, most of the p53^+^ (>85%) and p21^+^ (>92%) cells were myogenic independently of their genotype, as they expressed the lineage marker mGFP (Fig. 5a-f). In addition, the small fractions of p53^+^/mGFP^−^ cells and p21^+^/mGFP^−^ cells remained constant between both control and mutant muscles. Therefore, these results show that the absence of Numb/Numbl in the myogenic lineage (mGFP^+^) results in the cell autonomous induction of the senescent mediators p53 and p21. The findings reported here contrast with the previously described function of Numb in mammary cancer cells where Numb was reported to form a tripartite complex with p53 and the E3 ubiquitin ligase MDM2, hence preventing p53 ubiquitination and further degradation^37^. Our observations thus suggest that the regulation of p53 levels by Numb during the process of regeneration is indirect. We then determined whether p53 was sufficient to generate supernumerary SAβGal^+^ cells in *Numb* mutants by analysing *Numb:p53* compound mutants at 10 DPI. Strikingly, the number of SAβGal^+^ cells in mutants decreased to the levels observed in controls (Fig. 5g) as it was the case for anti-oxidant treatment indicated above. Most importantly, the muscle histological phenotype of *Numb* mutant TA muscle, including the increased fibrosis, was rescued in the absence of *p53* (Fig. 5h–j). We thus identify *p53* as a major regulator of a novel type of senescence induced by the absence of *Numb* during muscle regeneration.

## Discussion

Our study shows that cellular senescence participates in the process of skeletal muscle regeneration. We showed in control mice, that some endothelial cells (Flk-1^GFP/+^ cells) are senescent and that the induction of the SAβGal^+^ cells partially depends on *Ink4a/ARF* activity. Our findings together with the described beneficial action of senescent fibroblasts and endothelial cells during wound healing^13^ suggest that cellular senescence is a common cellular mechanism used during the process of regeneration of distinct organs.

The absence of Numb in the myogenic lineage induced a previously unreported type of senescence that acts in a cell autonomous manner. This persists when regeneration is virtually complete and it requires both p53 and elevated levels of ROS. Importantly, these factors clearly distinguished *Numb* mutant-specific senescence from the cellular senescence observed during organogenesis and wound healing that was reported to depend on p21 and p16/p21, respectively^11^,^12^,^13^. Instead, our studies suggest that *Numb* mutant-specific senescence is more closely related to oncogene-induced senescence models where p38 MAPK activity induced by elevated ROS regulates phosphorylation and activity of p53 (ref. 38). The ability to isolate the population of myogenic cells affected by the loss of *Numb* will allow the future identification of the underlying mechanisms initiating senescence when the function of Numb is compromised.

Furthermore, our findings underscore the importance of the communication between myogenic cells and their environment during regeneration. The rescue of the impaired regeneration phenotype in *Numb* mutant mice correlated with the loss of supernumerary SAβGal^+^ cells (Figs 4j, k and 5g–j). In addition, our *ex vivo* experiments lead us to propose that *Numb* mutant-specific senescent cells act in a paracrine manner to recruit macrophages and sustain inflammation, thereby promoting an increase in fibrosis. Chronic inflammation and aberrant fibrosis are also associated with numerous muscle pathologies. In this scenario, we show that in the absence of *Numb*, the phenotype of dystrophic mice is exacerbated. Therefore, close examination of the properties of activated satellite cells in relation to their environment, such as the identification of secreted factors, could be informative in devising therapeutic strategies for muscle pathologies.

## Methods

### Mice and breeding

Animals were handled as per European Community guidelines and the ethics committee of the Institut Pasteur (CTEA) approved protocols. *Tg:Pax7CT2* mice^24^ were used to delete *Numb* floxed allele in satellite cells. *Numb*^*F/F*^*:Numbl*^*F/F*^were obtained from Jackson Laboratories (005384; ref. 25). *Numb:Numbl* mutant mice were generated by crossing *Tg:Pax7CT2/+:Numb*^*F/+*^*: Numbl*^Δ*/+*^males with *Numb*^*F/+*^*:Numbl*^*Δ/+*^ females containing the reporter allele *R26*^*mT-mG*^ (Jackson 007576; ref. 26). All cells from *R26*^*mT-mG*^ mice are expected to express mTomato except those that have been Cre-recombined and become mGFP^+^. We previously generated a *Numbl* constitutive deleted allele by recombination of *Numbl* floxed allele in the germ line by crossing the *Numbl*^*F/F*^ mice with *PGK-Cre* mice^39^. The genetic background of the resulting mice was mixed (C57BL/6J and 129/Sv). During the course of the experiments we noticed that *Tg:Pax7CT2/+:Numb*^*F/F*^*: Numbl*^*Δ/Δ*^mice and *Tg:Pax7CT2/+:Numb*^*F/F*^*:Numbl*^*Δ/+*^mice exhibited the same phenotype (heterogenous fiber diameters, supernumerary SAβGal^+^ cells) suggesting that *Numbl* did not contribute to the observed impaired regeneration phenotype. Therefore, to address the function of p53 in *Numb* mutant we examined *Tg:Pax7CT2/+;Numb*^*F/F*^*;Numbl*^*Δ/+*^*;p53*^*−/−*^ (hereafter mentioned *Numb:p53*). For easy identification of Pax7^+^ cells, *Tg:Pax7nGFP* mice were used^40^. All experiments were carried out on males except females were also taken to examine *Numb:p53* compound mutants and control littermates. *DMD*^*mdx-βgeo*^, *p53*, *INK4a/ARF* mice were described previously^31^,^41^,^42^. *Flk-1*^*GFP/+*^ mice, in which the GFP is targeted in vascular endothelial growth factor–receptor 2 gene locus, were kindly provided by A. Medvinsky (Institute for Stem Cell Research, University of Edinburgh, UK).

### 4-OHT preparation and injection

Recombination of *loxP* sites was driven by intraperitoneal injections of 4-OHT(H6278 Sigma). Briefly 4-OHT was diluted^43^ to 40 mg ml^−1^ in 100% ethanol, then diluted in cremophor EL (C5135, Sigma) to 20 mg ml^−1^ and further diluted in 0.9% NaCl to 5 mg ml^−1^. 4-OHT solution was delivered by intraperitoneal injections (1 mg per 30 g); 9 injections were performed during 5 days for adult mice (6/8 weeks) and 1 injection every 5 days for perinatal mice (2 weeks).

### Injury and NAC treatment

Mice were anesthetised by intraperitoneal injection of a solution of 0.9% NaCl_2_, 0.5% Imalgene (Merial), 2% Rompun (Bayer; 100 μl per 25 g). TA muscles were injured by injection 20 μl of snake venom notexin (10 mg ml^−1^) or cardiotoxin (10 mM) (L8104 and L8102, Lotaxan Valence, France, www.latoxan.com). NAC (A9165 Sigma) was added to the drinking water from 5–10 DPI (1 g per 100 ml). The solution was changed every 2 days.

### Isolation of satellite cells and cell culture

TA muscle (for injured muscle) or muscles from the entire hindlimbs and forelimbs (for isolation of quiescent satellite cells) were dissected and placed into cold DMEM. Muscles were then chopped and put into a 50 ml Falcon tube containing 30 ml of DMEM (31966 Gibco), 0.1% Collagenase D (1088866 Roche), 0.25% trypsin (15090-046 Gibco), DNase 10 mg ml^−1^ (Roche 11284932001) at 37 °C under gentle agitation for 30 min. Digests were allowed to stand for 5 min at room temperature (RT) and the supernatants were collected on 5 ml of foetal bovine serum (FBS; Gibco) on ice. The digestion was repeated 3–5 times until complete digestion of the muscle. The supernatants were filtered through a 70-μm cell strainer (BD Falcon). Cells were spun for 15 min at 515 r.c.f. at 4 °C, the pellets were resuspended in 1 ml of DMEM 2% FBS and filtered through a 40-μm cell strainer (BD Falcon) before cell sorting. Cells were isolated based on size, granulosity and mGFP levels using a FACs MoFlo Legacy or Astrios (Beckmann Coulter). Cells were collected after sorting directly in culture media (20% FBS, 1% Penicillin-Streptomycin (15140 Gibco), 2% Ultroser G (15950-017 Pall Biosepra) in 50:50 DMEM:F12 (31966 and 31765 Gibco). Cells were plated at low density (3,000 cells cm^−2^) on glass coverslips that were coated successively with 10 mg ml^−1^ poly-D-lysine (P6407 SIGMA) and 1 mg ml^−1^ matrigel (354234 BD Biosciences) or directly on regular culture dishes coated with matrigel. For all experiments, control and mutant cells were plated at the same density.

The quantification of Pax7^+^ cells was performed from TA-injured muscles (activated cells) or diaphragm (quiescent cells) from *Tg:Pax7CT2/+;Tg:Pax7nGFP;Numb*^*F/+*^*;Numbl*^*Δ/+*^(control) and *Tg:Pax7CT2/+;Tg:Pax7nGFP;Numb*^*F/F*^*;Numbl*^*Δ/Δ*^(mutant) mice. Enumeration of GFP^+^ cells was performed with the 123 count eBeads (01-1234 eBiosciences) using a FACS Aria III (BD Biosciences).

### Isolation of macrophages and transwell assay

Muscles from forelimbs and hindlimbs of one mouse were chopped and divided into 4 × 50 ml Falcon tubes containing 30 ml of HBSS (24020-091 Gibco), 0.04% Collagenase A (11088793001 Roche), 0.3% Dispase II (04942078001 Roche), DNase 10 mg ml^−1^ (11284932001 Roche) at 37 °C under gentle agitation for 90 min. The supernatants were filtered through a 70-μm cell strainer. Cells were spun for 15 min, 515 r.c.f. at 4 °C, the pellets were resuspended in 1 ml of HBSS, 2% FBS containing mouse BD-FC, (clone 2.4G2, 553142 BD Pharmingen) and incubated on ice for 30 min. Five volumes of HBSS were added and cells were spun for 15 min, 515 r.c.f. at 4 °C. The pellets were resuspended in HBSS, 2% FBS containing the anti-mouse F4/80-APC (1/800, clone BM8, 17-4801-82 eBioscience) and the anti-mouse CD45-PeCy7 (1/200, clone 30F11, 25-0451-81 eBioscience) and incubated on ice for 30 min. Five volumes of HBSS were added and cells were spun for 15 min, 515 r.c.f. at 4 °C. The cells were resuspended in HBSS, 2% FBS and filtered through a 40-μm cell strainer before cell sorting. Macrophages were isolated based on size, granulosity and expression of CD45 and F4/80 using a FACS Aria III (BD Biosciences). CD45^+^/F4/80^+^ cells were collected in DMEM/F12 and plated on a 8 μM polycarbonate membrane (1,500 cells per 6.5 mm insert; 3422, Costar). The inserts were cultured in conditioned medium derived from mutant or control reserve cells. Conditioned medium was obtained from the medium of mutant and control reserve cells plated at the same density, cultured for 7 DIV and filtered through a 0.22-μm filter. After 6 h of culture, the upper surface of the insert was scraped to remove the cells that did not migrate, the membrane was fixed in 4% PFA 5 min and subsequently processed for immunostaining using the anti-F4/80 antibody (see below). The polycarbonate membrane was cut and mounted between slide and coverslip to allow counting of F4/80^+^/Hoechst^+^ cells under a microscope.

### Immunostaining and histology

For immunostaining, cells were fixed in PBS, 4% paraformaldhehyde (PFA; 15710 Electron Microscopy Sciences) 5 min at RT, washed in PBS and permeabilized with PBS, 0.5% Triton X-100 5 min at RT. After three washes in PBS, cells were blocked with PBS, 20% goat serum 1 h at RT. Primary antibodies were added to cells in PBS, 2% goat serum for 2 h at RT under gentle rocking. Cells were washed three times with PBS then incubated with the secondary antibodies 1 h at RT. Before mounting, cells were washed three times with PBS.

TA muscles were isolated from mice and frozen directly in isopentane for <1 min, then stored at −80 °C or directly cryosectioned (8 μM sections). For histology, the sections were kept at RT overnight before staining. Sections were then rehydrated in PBS for 10 min and fixed in 10% formalin for 3 min. Sections were then routinely stained with haematoxylin and eosin, Sirius Red or Von Kossa. For immunostaining the sections were fixed in PBS, 4% PFA at RT 10 min, washed 3X 10 min in PBS, incubated with the primary antibody in a solution of PBS, 10% foetal calf serum (FCS), 3% BSA, 0.5% Triton X-100 ON at 4 °C. Sections were washed in PBS, 0.5% Tween-20, 3X 10 min and incubated with the secondary antibody in the same solution as for the primary antibody 1 h at RT. Sections were washed in PBS, 0.5% Tween-20, 2X 10 min, once in PBS 10 min and mounted.

### Antibodies

Primary antibodies used include: anti-GFP (1/2,000, chicken polyclonal, Abcam ab13970); anti-Pax7 (1/20, mouse monoclonal, DSHB); anti-Calcitonin receptor (1/100, rabbit polyclonal, Serotec AHP 635); anti-HP1γ (1/2,000, mouse monoclonal, Euromedex, 2MOD-1G6-AS); anti-Myosin Heavy Chain (1/200, rabbit polyclonal, kindly provided by G. Cossu); anti-Ki67 (1/300, rabbit polyclonal, Abcam 15,580); anti-p53 (1/500, rabbit polyclonal, Leica, MC5); anti-p21 (1/2, rat monoclonal, CNIO HUGO291); anti-Tcf4 (1/100, rabbit monoclonal, Cell Signalling C48H11); anti-F4/80 (1/50, rat monoclonal, Serotec MCA 497). Alexa-conjugated secondary antibodies (1/500, Molecular Probes) together with 1 μg ml^−1^ of Hoechst-33342 were used for immunofluorescence. Following immunofluorescence, cells and sections were mounted with VECTASHIELD Mounting Media between slide and coverslip. Immunohistochemistry was performed by incubation with Biotin-conjugated secondary antibodies (1/500, Jackson laboratories), followed by incubation with Peroxidase (HRP) Polymer conjugated streptavidin (1/2,000, S2438 Sigma). Peroxidase activity was detected using the DAB Peroxidase Substrate Kit (SK-4100 Vector).

### SAβGalactosidase

Cells and sections were fixed for 4 min at RT in a solution of PBS, 1% PFA, 0.2% glutaraldehyde. Samples were washed in PBS pH7 2X 10 min and incubated for 30 min in PBS pH6 and further incubated in an X-gal solution (4 mM K_3_Fe(CN)_6_, 4 mM K_4_Fe(CN)6, 2 mM MgCl_2_, 0.02% NP-40 (Igepal) and 400 μg ml^−1^ X-gal (15520-018 Sigma) in PBS pH6) at 37 °C ON for cells and 2X 24 h for sections. For sections, X-gal substrate was changed after 24 h. Samples were washed in PBS, and post-fixed in 1% PFA 5 min for cells and 30 min for sections. After washes, 3X for 10 min in PBS, samples were mounted in PBS, 20% glycerol or processed for immunochemistry.

### RT-qPCR

Total RNA was extracted from cells isolated by FACS directly into cell lysis buffer (RLT; Qiagen RNeasy Micro Kit). cDNA was prepared by random-primed reverse transcription (Super Script III, 18080044 Invitrogen) and real-time PCR was performed using SYBRGreen Universal Mix (13608700 Roche).

Primers used include:

*Col1a1*: fw 5′-CCCTGGTCCCTCTGGAAATG-3′; rv 5′-GGACCTTTGCCCCCTTCTTT-3′;

Il6: fw 5′-ATGCTCCCTGAATGATCACC-3′; rv 5′-TCACAGATGGCGTTGACAAG-3′;

*TBP*: fw 5′-ATCCCAAGCGATTTGCTG-3′; rv 5′-CCTGTGCACACCATTTTTCC-3′.

### Statistics and quantifications

All experiments were carried out on a minimum of 3 mice except where stated otherwise (see Figure legends). No statistical method was used to predetermine sample size, no animals were excluded from the analysis and the experiments were not randomized. The investigators were not blinded to allocation during experiments and outcome assessment. The graphs were plotted and statistical analysis was performed using GraphPad Prism Software. All data points are presented as mean±s.e.m. (error bars) except where stated otherwise (see Figure legends). Mann-Whitney test (two-tailed) was applied in all cases (non significant (NS) *P*≥0.05; *P<0.05; **P<0.01; ***P<0.001). When quantifications are given per unit area, one unit area is defined by a surface of 75,848.74 μm^2^ (corresponding to a × 40 field acquired with a confocal Leica Spe microscope). A correction index has been used for the bright field pictures taken with a Nikon Eclipse microscope. A minimum of 10 fields/TA muscle section have been quantified for each marker after immunofluorescence and 40 fields/TA muscle section after SAβGal and Sirius red stainings.

Quantification of fibrosis was performed from Sirius Red staining using ImageJ Software. After substraction of the background from the original picture, the picture was converted to a binary picture. The list on the histogram gives minimum and maximum values corresponding to the area of fibrosis and the area devoid of fibrosis, respectively. Control and mutant sections from one given condition were processed for staining and quantification at the same time.

## Additional information

**How to cite this article:** Le Roux, I. *et al*. Numb is required to prevent p53-dependent senescence following skeletal muscle injury. *Nat. Commun.* 6:8528 doi: 10.1038/ncomms9528 (2015).

## Supplementary Material

Supplementary Information Supplementary Figures 1-4, Supplementary Methods and Supplementary References
