# Supplementary material for: Numb is required to prevent p53-dependent senescence following skeletal muscle injury
Source: Nat Commun. 2015 Oct 27;6:8528. doi: 10.1038/ncomms9528 (PMC4639798; doi:10.1038/ncomms9528)
Supplement: Supplementary Information — Supplementary Figures 1-4, Supplementary Methods and Supplementary References [file ncomms9528-s1.pdf]

Supplementary Figure 1

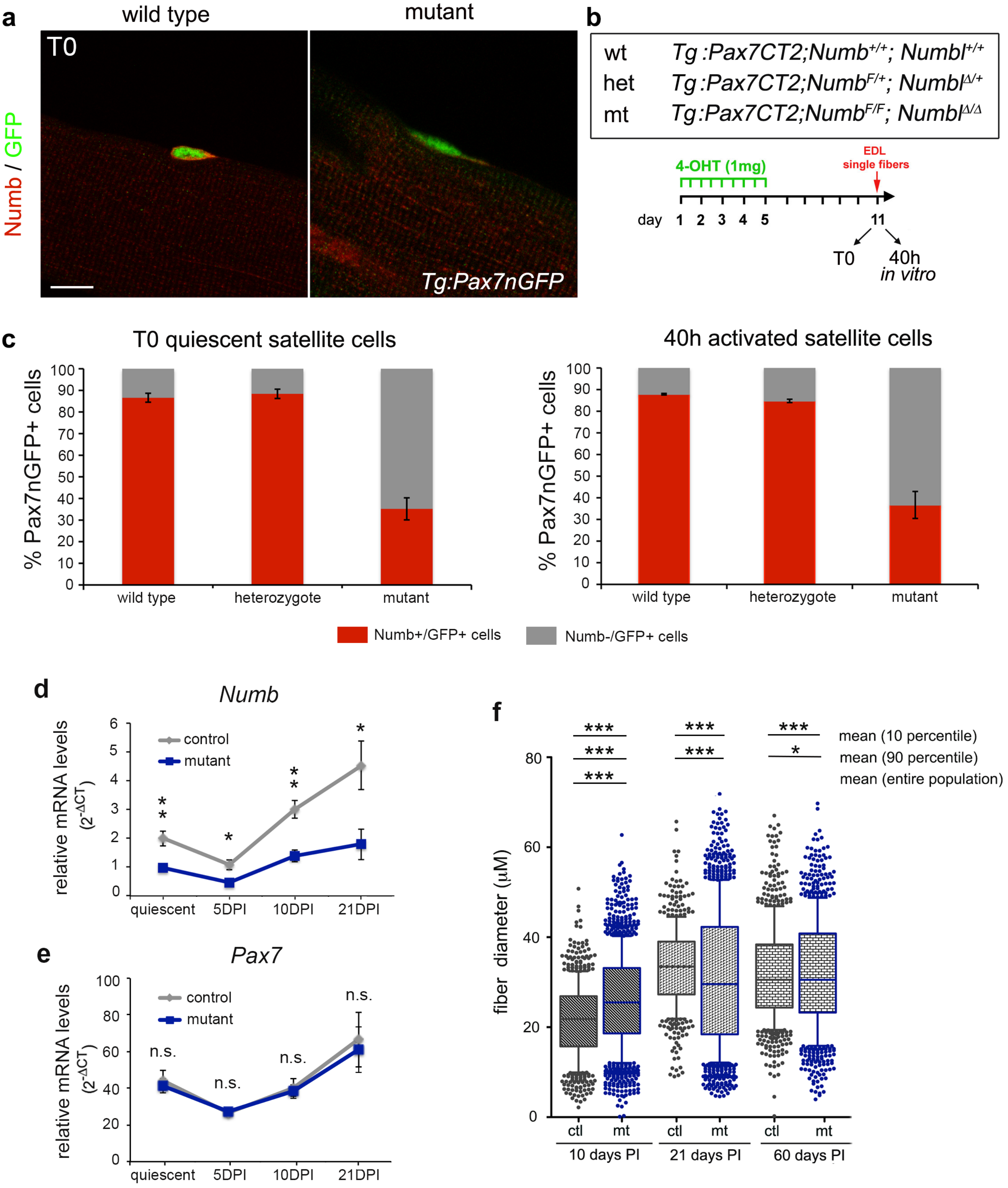

Supplementary Figure 1.

Deletion of *Numb:Numbl* in Pax7 lineage affects muscle regeneration.

(**a**) Immunofluorescence on freshly dissociated single myofibers from *Tg:Pax7nGFP extensor digitorum longus* (EDL) muscle using anti-Numb and anti-GFP antibodies. Scale bar 10μM. (**b**) Top: genetic combinations used in the study. Bottom: scheme of strategy used to delete *Numb* floxed allele. (**c**) Quantification of Numb+ cells in quiescent (T0) and activated (40h in culture) among the Pax7nGFP+ cells on fibers from wild type (T0: n=3 mice, 86.6±2.1 cells; 40h: n=2 mice, 87.9±0.36 cells), heterozygous (T0: n=2 mice, 88.4±2.2 cells; 40h: n=2 mice, 84.8±0.77 cells) and mutant (T0: n=5 mice, 35.2±5.1 cells; 40h: n=3 mice, 36.7±6.3 cells). Of note, early stage of proliferation of mutant satellite cells was not affected as the proportion of Pax7+/Numb- vs. Pax7+/Numb+ cells was maintained in mutants from the time of isolation to 40h of culture (about 2-3 cell cycles). Heterozygous mice (*Tg:Pax7CT2 ;*$Numb^{F/+}$*;*$Numbl^{\Delta/+}$) expressed Numb at similar intensity and in similar number of cells compared to wild type mice (*Tg:Pax7CT2;*$Numb^{+/+}$*;*$Numbl^{+/+}$). Controls are either wild type or heterozygous mice. (**d**, **e**) Relative levels of *Numb* and *Pax7* mRNA ($2^{-\Delta CT}$) before and during regeneration time. TBP was used as reference gene. Quiescent stage, control: n=9 mice and mutant: n=8 mice; 5 Days post in jury (DPI), control: n=4 mice and mutant n=5 mice; 10 DPI, control n=8 mice and mutant n=7 mice; 21 DPI, control n=5 mice and mutant n=6 mice. Mann Whitney (MW) test control vs mutant: *Numb* quiescent stage, p=0.0016; *Numb* 5DPI, p=0.0159; *Numb* 10DPI, p=0.0022; *Numb* 21DPI, p=0.0303; *Pax7* quiescent stage, p=0.8884; *Pax7* 5DPI, p=0.9048; *Pax7* 10DPI, p>0.9999; *Pax7* 21DPI, p=0.9307. Quantifications are presented as mean ± SEM. **(f)** Quantification of mGFP+ fiber diameter 10, 21 and 60 DPI in mutants (5, 4, 3 mice; respectively) compared to controls (2, 3, 3 mice; respectively). Boxes represent total population of fiber diameters; lines highlight mean and standard deviation. Dot plots represent 10 and 90 percentiles (10% smallest and 10% largest fibers; respectively). Mean fiber size is significantly distinct between controls and mutants 10 DPI, MW p<0.0001 (mean fiber size, control: 10 DPI 21.51±8.4μM; 21 DPI 33.7±8.4μM; 60DPI 32±10.7μM; mutant: 10 DPI 26.2±10.7μM; 21 DPI 31±15μM; 60DPI 32±12.3μM). Ten, 21 and 60 DPI, the 10 percentiles are significantly distinct between controls and mutants (mean of 10 percentile, control, 10 DPI 7.2±1.8μM; 21 DPI 17.5±3.7μM; 60 DPI 15.7±3.5μM; mutant: 10 DPI 8.6±2.5μM; 21 DPI 9.1±2μM; 60 DPI 12±3; MW 10DPI p<0.0001, 21DPI p<0.0001, 21DPI p<0.0001). Ten, 21 and 60 DPI, the 90 percentiles are significantly distinct between controls and mutants

(Mean of 90 percentile, control: 10 DPI 36.4± 3.8μM, 21 DPI 50±4.7μM, 60 DPI 53.4±5.5μM; mutant: 10 DPI 49.2±3.7μM, 21 DPI 58.3±4.4μM, 60 DPI 54.3±4.4μM; MW 10DPI, $p<0.0001$; 21DPI, $p<0.0001$; 60DPI, $p=0.0438$).

Supplementary Figure 2

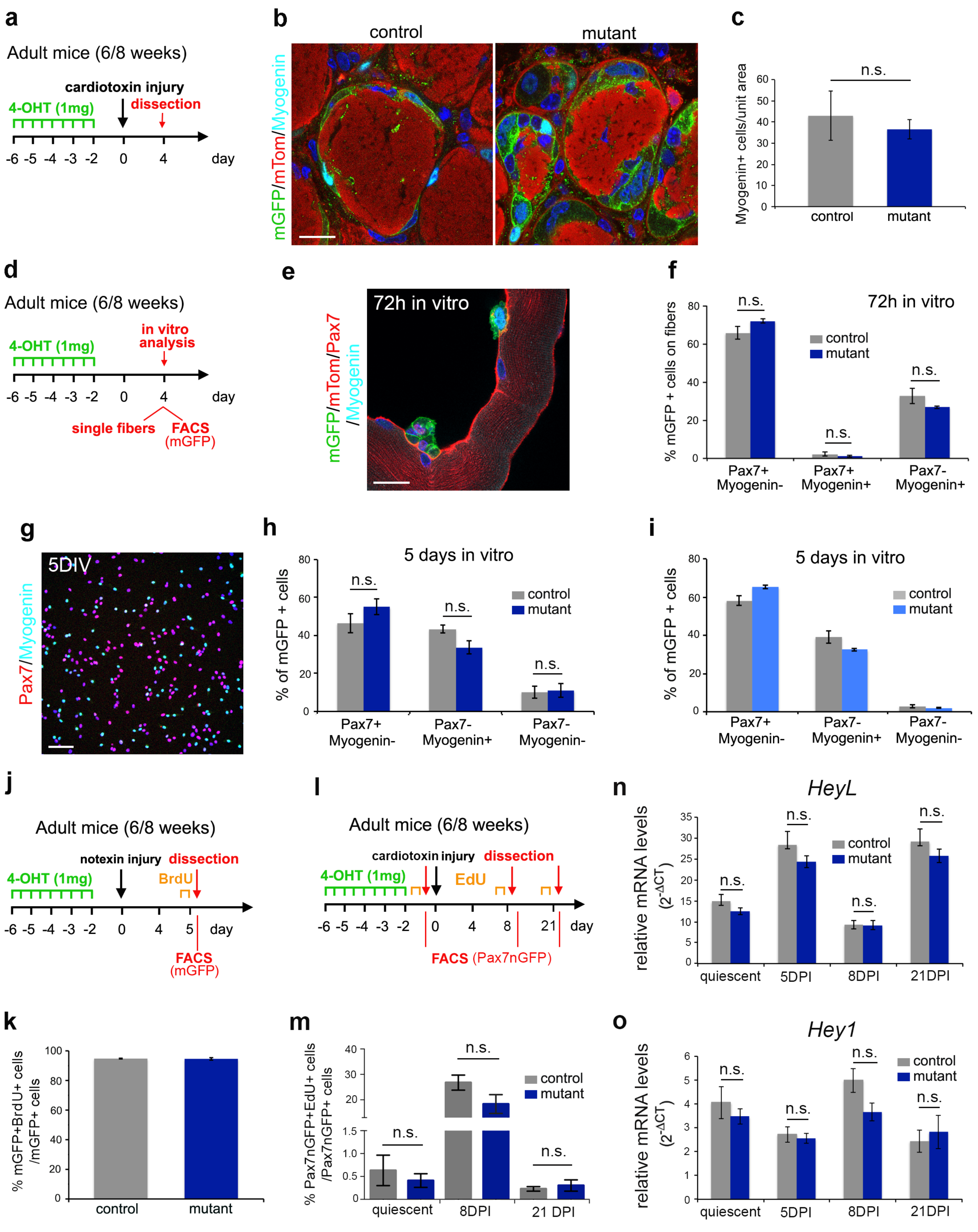

Supplementary Figure 2.

Differentiation and proliferation potentials of *Numb:Numbl* mutant satellite cells are not overtly compromised.

(**a**) Strategy used to investigate differentiation *in vivo* at 4 DPI. (**b**) Immunofluorescence on transverse sections with anti-GFP and anti-Myogenin antibodies. (**c**) The number of Myogenin+ (or differentiating) cells in controls and mutants was similar at 4 DPI (control: n=3 mice, 43±11.5 Myogenin+ cells/unit area; mutant: n=3 mice, 36.6±4.6 Myogenin+ cells/unit area). (**d**) Strategy used to investigate differentiation *ex vivo*. (**e**), Representative image of an isolated EDL fiber cultured for 72h and stained with anti-GFP, anti-Pax7 and anti-Myogenin antibodies. (**f**) Number of Pax7+ and Myogenin+ cells in mutants and controls after 72h in culture (control: n=4 mice, 65.8±3.4% Pax7+/Myogenin-/mGFP+ cells; 2.23±0.97% Pax7+/Myogenin+/mGFP+ cells; 32.7±4.1% Pax7-/Myogenin+/mGFP+ cells. mutant: n=3 mice, 72±1.2% Pax7+/Myogenin-/mGFP+ cells; 1.16±0.64% Pax7+/Myogenin+/mGFP+ cells; 26.9±0.7% Pax7-/Myogenin+/mGFP+ cells). (**g**) Representative image of isolated satellite cells cultured 5 DIV and immunostained with the anti-Pax7 and anti-Myogenin antibodies. (**h**, **i**) Quantifications of Pax7+ and Myogenin+ cells after 5 DIV isolated from either the *Tg:Pax7CT2* deleter mice ((**h**) control: n=3 mice, 46.5±5% Pax7+/Myogenin- cells; 43.3±2% Pax7-/Myogenin+ cells; 10.2±3.2% Pax7-/Myogenin- cells. Mutant: n=3 mice, 55±4.2% Pax7+/Myogenin- cells, 33.7±3.5% Pax7-/Myogenin+ cells; 11±3.5 Pax7-/Myogenin- cells) or the constitutive $Mrf4^{Cre}$ deleter mice in which all the mGFP+ cells are depleted for Numb ((**i**) control: n=2 mice, 58.1±2.5% Pax7+/Myogenin- cells; 39.1±3.3% Pax7-/Myogenin+ cells; 2.83±0.76 Pax7-/Myogenin- cells. Mutant: n=3 mice, 65.4±0.87% Pax7+/Myogenin- cells; 32.53±0.68% Pax7-/Myogenin+ cells; 2.06±0.29% Pax7-/Myogenin- cells). (**j**, **l**) Strategies used to assess proliferation *in vivo* during homeostasis and at 5, 8, and 21 DPI. Mice were injected with BrdU (50μg/g) or EdU (24μg/g) 24h and 18h before sacrifice. (**k**) Quantification of BrdU+/mGFP+ cells at 5 DPI in control (n=2 mice; 94.8±0.3 % BrdU+/mGFP+ cells) and mutant (n=2 mice; 94.6±0.8% BrdU+/mGFP+ cells). (**m**) Quantification of EdU+/Pax7nGFP+ cells. Quiescent cells, control: n=8 mice, 0.6±0.3% EdU+/Pax7nGFP+ % cells; mutant: n=7 mice, 0.4±0.1% EdU+/Pax7nGFP+. Activated cells at 8 DPI, control: n=8 mice, 26.8±3 EdU+/Pax7nGFP+; mutant n=6 mice, 18.3±3.7 EdU+/Pax7nGFP+. Cells at 21 DPI, control: n=3 mice, 0.2±0.05% EdU+/Pax7nGFP+; mutant n=4, 0.3±0.1% EdU+/Pax7nGFP+. (**o**, **p**) Relative levels of *HeyL* and *Hey1* mRNA ($2^{-\Delta CT}$) before and during

regeneration. TBP was used as reference gene. Quiescent stage, control *HeyL*: n=9 mice and mutant *HeyL*: n=8 mice; control *Hey1*: n=6 mice and mutant *Hey1*: n=5 mice. 5 DPI, control: n=4 mice and mutant n=5 mice; 10 DPI, control n=8 mice and mutant n=7 mice; 21 DPI, control n=5 mice and mutant n=6 mice. Quantifications are presented as mean ± SEM except for (**i**) where quantifications are presented as mean ± SD. Scale bars, **b**,**e**: 40μM, **g**: 100μM.

Supplementary Figure 3

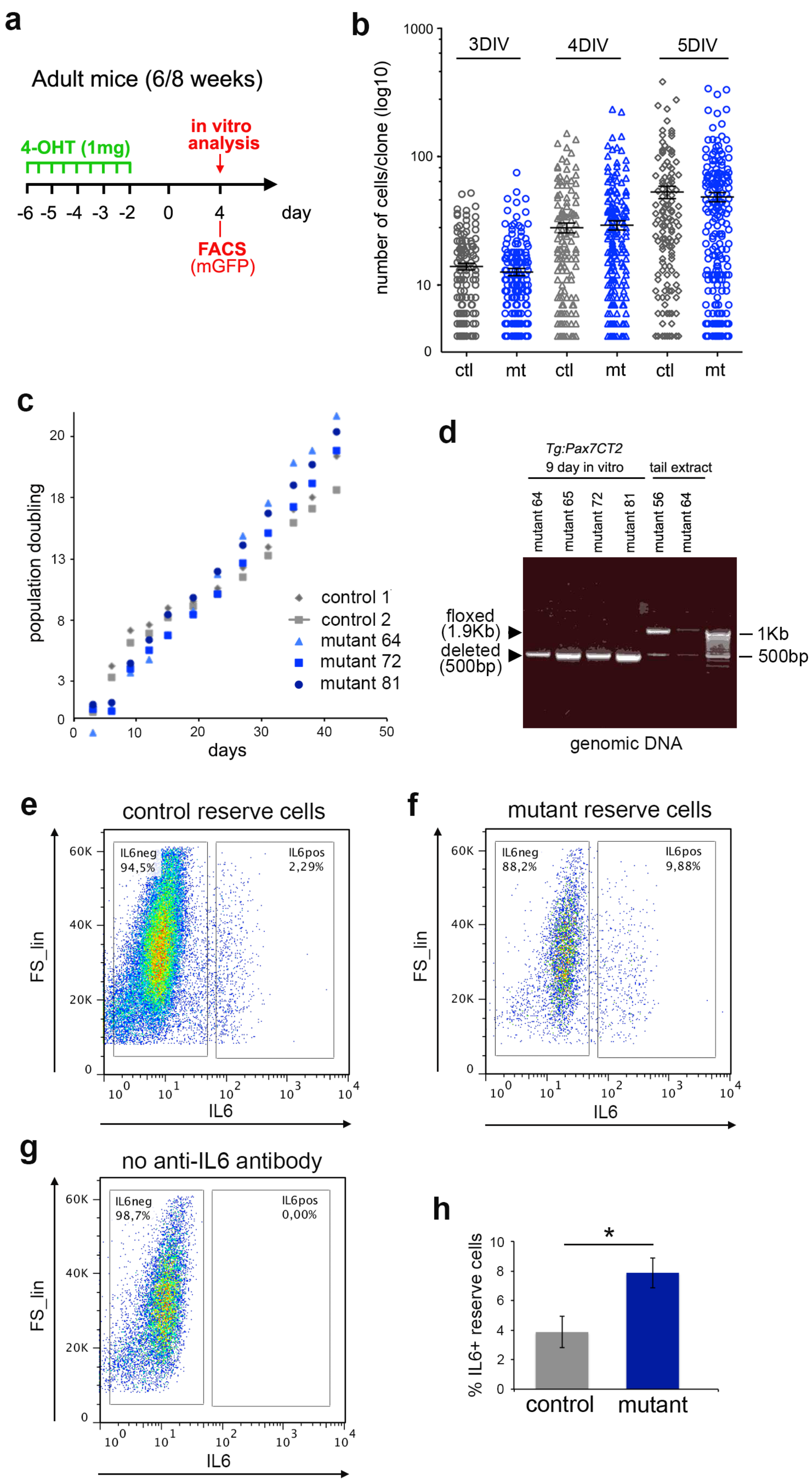

Supplementary Figure 3.

Properties of *Numb:Numbl* mutant primary and reserve satellite cells.

(**a**) Strategy used to investigate proliferation *ex vivo* at single cell level. Single sorted cells were directly plated into 96 well plates. The cloning efficiency of control and mutant cells was similar demonstrating no change in cell survival between genotypes (control: n=2 mice; 72±2.2; mutant: n=3 mice, 73.8±7.8). (**b**) Distributions of clone size are alike between mutant and control cells 3, 4 and 5 DIV. Bars represent the mean of the clone size ± SEM (3 DIV control: n=137 clones, 13.9±0.9 cells/clone, mutant: n=168 clones, 12.6±0.8; 4 DIV control: n=143 clones, 27.9±2.5, mutant: n=198 clones, 29.2±2.4; 5 DIV control: n=133 clones, 53.0±5.9, mutant: n=197 clones, 48.5±4.0). (**c**) In parallel to the preparation of reserve cells, freshly isolated mGFP+ cells isolated from control or mutant mice were plated at low density, dissociated, counted and re-plated at low density every 3 to 4 days up to 12 times. Population doublings of control and mutant cells were calculated during 42 days in culture. Y-axis represents the cumulative plot of population doublings. (**d**) PCR performed on genomic DNA isolated from the cells after 9 days *ex vivo* (third passage), showed that in mutants the majority of cells had deleted the *Numb* allele compared to crude DNA extract from a mixed population of cells taken from a tail biopsy. Kb: Kilobase pair; bp: base pair. Note that cells still expressing *Numb* in mutant condition did not have a proliferative advantage compared to *Numb* depleted cells following Cre-recombinase activity. (**e**, **f**) FACS plots of mGFP+ control and mutant reserve cells immunostained with an anti-IL6 antibody. (**g**) FACS plot of reserve cells incubated uniquely with the secondary antibody used to label anti-IL6 in (**e**) and (**f**). (**h**) Quantification of the percentage of reserve cells cultured for 9 DIV and expressing IL6 (control: n=6, 3.9±1 % IL6+ cells; mutant n=8 mice, 7.9±1 IL6+ cells; MW p=0.0293). Quantifications of IL6 are presented as mean ± SEM.

Supplementary Figure 4

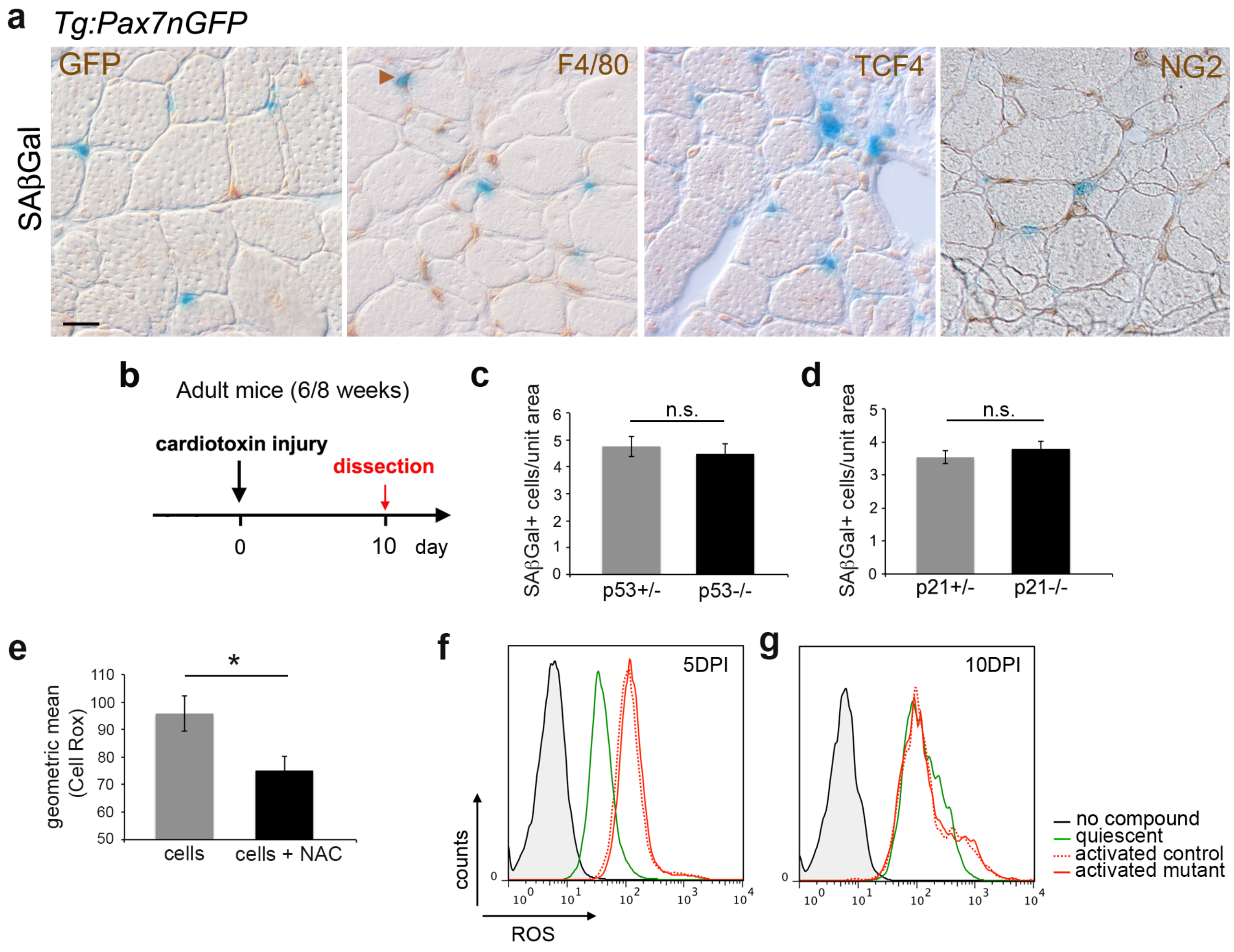

Supplementary Figure 4.

Senescence and ROS levels during muscle regeneration.

(**a**) SAβGal staining on *Tg:Pax7nGFP* TA muscle 10 DPI combined with immunohistochemistry using anti-GFP, anti-F4/80, anti-TCF4, or anti-NG2 antibodies. Note, some SAβGal+ cells expressed F4/80 (brown arrowhead) but the majority of macrophages were SAβGal negative. Data are representative of 3 independent *Tg:Pax7nGFP* mice. (**b**) Scheme of the experiment. Data were collected from transverse TA muscle cryosections of adult mice. Scale bar 50μM. (**c**, **d**) Quantification of SAβGal+ cells present at 10 DPI, in *p53* and *p21* mutants and their control littermates (p53+/-: n=6 sections, 3 mice, 4.76±0.37 cells/unit area; p53-/-: n=6 sections, 3 mice 4.48±0.35 cells/unit area; p21+/-: n=6 sections, 2 mice, 3.54±0.19 cells/unit area; p21-/-: n=8 sections, 2 mice, 3.78±0.25 cells/unit area). (**e**) Treatment with NAC from 5-10 DPI decreased significantly ROS levels (Cell Rox signal) of Pax7nGFP+ cells at 10DPI (no treatment: n=6 mice, 95.9±6.4 geometric mean; NAC treatment: n=6 mice, 75.1±5.3 geometric mean; MW p=0.0411). (**g**, **h**) FACS histogram of quiescent and activated control and mutant mGFP+ cells incubated with Cell Rox. Activated control and mutant cells at both time points display similar amounts of ROS (geometric mean of activated mGFP+ cells (mean ± SD), 5 DPI: control n=2 mice, 106.2±18.2; mutant n=2 mice, 133.5±3.5; 10 DPI: control n=3 mice, 146.7±9.1; mutant n=3 mice, 159±18.9). n.s., not significant.

Supplementary methods

Mice

Mrf4^{Cre} and *p21* mice were described[1], [2].

Fiber diameter

mGFP+ fiber diameter was measured from images acquired on a Leica Spe confocal. The small diameter of the fiber was quantified using ImageJ Software. A minimum of 170 fiber diameters was measured per mouse. The n-value used for statistics represents the number of fiber diameters per condition.

Single fiber preparation

Single fibers were isolated as described[3]. EDL muscles were dissected from adult mice (6-8 weeks old) and incubated for 1h in DMEM 0.2% collagenase (C-0130 Sigma) at 37°C. Following collagenase treatment, muscles were transfered to DMEM (31966 Gibco®) and fibers were mechanically dissociated by successive (<10) flushing using an eroded glass pasteur pipette. Fibers were cultured in 20% FBS, 1% Penicillin-Streptomycin in 50:50 DMEM:F12 at 37°C in 5%CO_2, 20%O_2.

RT-qPCR

Total RNA was extracted from cells isolated by FACS directly into cell lysis buffer (RLT; Qiagen RNeasy Micro Kit). cDNA was prepared by random-primed reverse transcription (Super Script III, 18080044 Invitrogen) and real-time PCR was performed using SYBRGreen Universal Mix (13608700 Roche).

Primers used include :

Numb45	fw TGGCAGACAGATGAAGAAGG	rv CACAGATGTGCATTCCTCTTG
Pax7	fw GACAAAGGGAACCGTCTGGAT	rv TGTGAACGTGGTCCGACTG
HeyL	fw TATGATCCCTCTGCGCTTCT	rv CATCGATGTGGGTCAAGAGA
Hey1	fw CGGACGAGAATGGAAACTTGAG	rv CAAAACCTGGGACGATGTCG

Immunostaining and EdU staining

For immunostaining, isolated myofibers were fixed in PBS, 4% PFA for 5 min. Fixed myofibers were washes in PBS 3 times and then permeabilised in a solution of 20 mM HEPES, 300 mM sucrose, 50 mM NaCl, 3 mM $MgCl_2$ and 0.5% Triton X-100 (pH 7) at 4°C for 15 min as described by [4]. Fibers were washed in PBS 3 times. Primary antibodies were

added in PBS, 2% GS, 0.01% Triton-X100 ON at 4°C. Fibers were washed in PBS 3 times and myofibers were incubated 1 h at RT with secondary antibodies in the same solution as for the primary and mounted after 3 washes in PBS.

Antibodies used include: anti-Numb (1/200, rabbit polyclonal)[5]; anti-Myogenin (1/100, rabbit polyclonal, Santa-Cruz (m-225) sc-576); anti-BrdU (1/100, mouse monoclonal, 347580 BD); anti-IL6 (1/100, rat monoclonal, clone MP5-20F3, 14-7061-85 eBiosciences); anti-NG2 (1/100, rabbit, NAB5320 Chemicon Millipore). EdU staining was chemically revealed with the Click-iT kit (C10640 Life Technologies) and analysed after FACS (CyAn ADP ; Beckman Coulter) using Flow Jo Software.

Population doublings, clonal analysis

Population doubling (PD) per passage was calculated as $\log_2$ (number of cells at time of subculture/number of cells plated). Cumulative PD was plotted against total time in culture to determine replicative life span. For clonal analysis, the acquisitions of Hoechst staining were conducted using the high content screening OPERA (Perkin Elmer) and the quantification of the nuclei were performed using the Acapella® software (Perkin Elmer).

ROS quantification

The fluorescent probe CellROX deep Red Reagent (C10422 Life technologies) was used to monitor the intracellular generation of ROS. Cells were incubated in DMEM 2% FCS with CellROX deep red 30 min at 37 °C and fluorescence intensity was measured by FACS (CyAn ADP ; Beckman Coulter). Data were analysed using Flow Jo Software.

Supplementary references

1. Keller, C., Hansen, M.S., Coffin, C.M. & Capecchi, M.R. Pax3:Fkhr interferes with embryonic Pax3 and Pax7 function: implications for alveolar rhabdomyosarcoma cell of origin. *Genes & development* **18**, 2608-2613 (2004).
2. Deng, C., Zhang, P., Harper, J.W., Elledge, S.J. & Leder, P. Mice lacking p21CIP1/WAF1 undergo normal development, but are defective in G1 checkpoint control. *Cell* **82**, 675-684 (1995).
3. Gayraud-Morel, B. *et al.* A role for the myogenic determination gene Myf5 in adult regenerative myogenesis. *Developmental biology* **312**, 13-28 (2007).
4. Collins-Hooper, H. *et al.* Age-related changes in speed and mechanism of adult skeletal muscle stem cell migration. *Stem Cells* **30**, 1182-1195 (2012).
5. Jory, A. *et al.* Numb promotes an increase in skeletal muscle progenitor cells in the embryonic somite. *Stem cells* **27**, 2769-2780 (2009).
